# Morbidity and Mortality after Cytoreductive Surgery and HIPEC in a National Reference Center: A Six-Year Experience under Independent Evaluation

**DOI:** 10.3390/jcm13175182

**Published:** 2024-08-31

**Authors:** Miguel Enrique Alberto Vilchez, Sebastian Halskov, Axel Winter, Johann Pratschke, Beate Rau, Safak Gül

**Affiliations:** 1 Surgical Department, Charité—Universitätsmedizin Berlin, Campus Virchow Klinikum, 13353 Berlin, Germany; miguel.alberto@charite.de (M.E.A.V.); beate.rau@charite.de (B.R.); 2 Department of Radiology, Charité—Universitätsmedizin Berlin, 10117 Berlin, Germany

**Keywords:** peritoneal surface malignancies, postoperative complications, CRS, HIPEC, Clavien–Dindo

## Abstract

**Introduction:** Cytoreductive surgery (CRS) coupled with hyperthermic intraperitoneal chemotherapy (HIPEC) is a potentially curative approach to peritoneal disease (PD) and corresponds to the needs of patients with dire survival rates. However, the oncological community remains cautious toward this procedure because of its significant morbidity and mortality rates. This evolving evidence around CRS and HIPEC and its impact suggests a need for more standardized procedures in existing centers. Because of its complexity and potential for high morbidity and mortality, critical observation of our center’s complication rates using complication management documentation tools were crucial to further develop our standard operating procedures (SOP) and maximize patient safety. **Methods:** Our prospectively maintained institutional database was queried to identify all patients who underwent CRS and HIPEC and had a filled-out quality management (QM) and complication management documentation tool at discharge at the surgical department of the Charité—Universitätsmedizin Berlin, Campus Virchow Klinikum, Berlin, Germany, between January 2018 and December 2023. **Results:** A total of 155 patients had a surgical and/or medical complication recorded. In total, 305 surgeries were surveyed. Some patients had more than one postoperative complication; hence, 344 events in 50 categories were recorded, of which 267 were graded 3a or higher in 92 patients. The most common medical complications were of pulmonary and renal origin. On the surgical side, surgical site infections (SSI) were most common. The incidence of anastomotic leakage (AL) was 5% (*n* = 8), with no events between 2021 and the present. Patients with longer surgery duration times were at higher risk for developing postoperative complications. **Conclusions:** Major abdominal surgeries like CRS and HIPEC are associated with significant patient morbidity despite achieving optimal oncological outcomes. Postoperative complications are managed through strict surveillance and transparency, particularly in our large reference centers, to minimize patient risk. Quality management programs in our department have successfully maintained high standards of care without compromising patient safety.

## 1. Introduction

A comprehensive treatment of peritoneal disease (PD) requires excellent detailed knowledge of the patient’s clinical history and a multimodal and interdisciplinary approach. Surgical treatment of both malignant and benign diseases of the peritoneum requires special surgical skills and experience and outstanding perioperative care. Malignant peritoneal disease is a frequent progression of gastrointestinal or gynecological cancers; nevertheless, there are also cancers of the peritoneum itself, like peritoneal mesothelioma and primary peritoneal cancer. An extensive, aggressive, and surgically radical treatment is necessary to improve prognosis and quality of life (QOL). Even benign diseases of the peritoneum need aggressive treatment due to rapidly decreasing QOL. Cytoreductive surgery (CRS) coupled with intraperitoneal chemotherapy (IP), in particular hyperthermic intraperitoneal chemotherapy (HIPEC), emerged as a potentially curative approach to PD and corresponds to the needs of patients with dire survival rates. However, the oncological community remains cautious toward this procedure because of its significant morbidity (18–43%) and mortality rates (up to 10%) [1,2,3]. Although important discrepancies in reporting and interobserver variability may cause these rates to be heterogenous, it is true that CRS and HIPEC is a major abdominal surgery with the intraperitoneal application of chemotherapeutics, where relatively high rates of complication and toxicity are to be expected.

Nevertheless, this procedure is a recognized treatment for pseudomyxoma peritonei and shows increasing evidence of providing benefits for patients with PD of malignant gastrointestinal and gynecological origin, as well as peritoneal mesothelioma. This evolving evidence around CRS and HIPEC and its mainly positive impact demands a need for standardized procedures in existing reference centers. Taking into consideration the complexity and high morbidity of this procedure, our aim was to have a critical look into our postoperative complications making use of a tailored documentation tool anchored in our hospital information system and scrutinized by our colleagues, thereby gaining further knowledge to mature our standard operating procedures (SOP) and maximize patient safety.

## 2. Methods

### 2.1. Study Population

**Figure 1:**
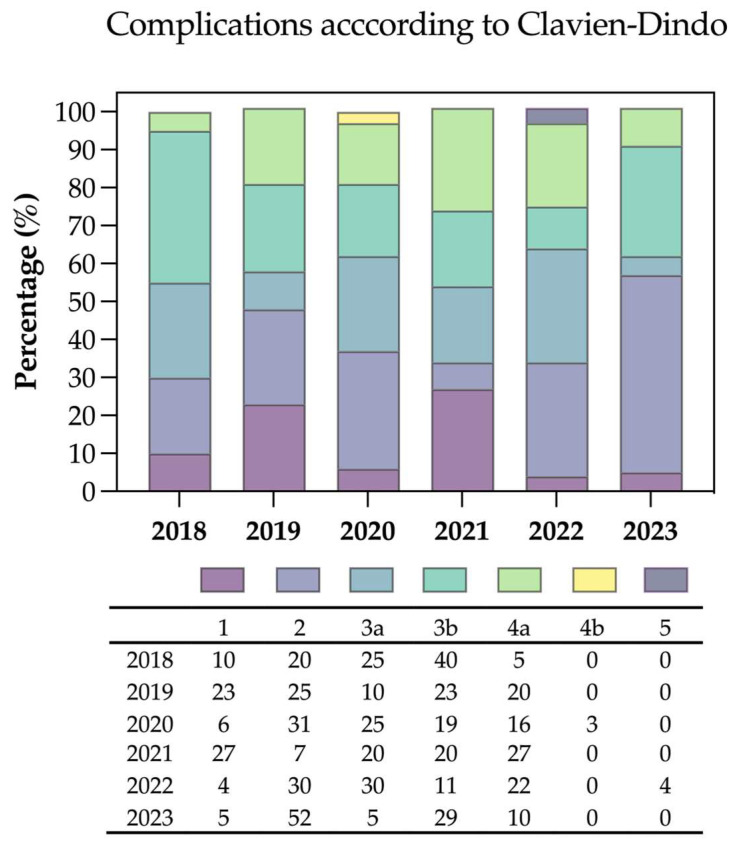
Percentage of complications per year (Clavien–Dindo). The percentage shown in the table of the figure represents a percentage in relation to the total number of complications in that year. *N* = 155 patients; *N* total patients per year: 2018: 44, 2019: 70, 2020: 55, 2021: 34, 2022: 58, 2023: 44.

We carried out a retrospective single-center observational study where our prospectively maintained institutional database was queried to identify all patients who underwent CRS and HIPEC for peritoneal disease and had a filled-out quality management (QM) and complication management documentation tool (please see reference Winter et al. [4]—Figure 1) from the physician in charge of discharge at the surgical department of the Charité—Universitätsmedizin Berlin, Campus Virchow Klinikum, Berlin, Germany, between January 2018 and December 2023. Only patients that complied with both criteria were included in the study. We created a sub-query with the following data: gender; age at time of surgery; duration of hospitalization, including time in the intensive care unit (ICU); origin of the primary tumor; date of first diagnosis; metachronous or synchronous metastasis; peritoneal carcinomatosis index (PCI); completeness of cytoreduction (CC-) score; extent of surgery; type of HIPEC and characteristics; post-operative morbidity and mortality; type of complication management; and re-operation and re-admission to the intensive care unit (ICU). Our hospital complies with all requirements to be a certified center from the competent German authorities for the management of peritoneal disease, and as such, serves as a national reference center. Institutional ethical approval (EA4/238/21) was received for the study, and all patients were subject to informed consent.

### 2.2. Cytoreductive Surgery and HIPEC

In brief, all patients with PD were presented in our multidisciplinary tumor board (MDT) and were approved for CRS and HIPEC. Under general anesthesia and hemodynamic monitoring, a diagnostic laparoscopy was performed to assess the preliminary PCI score (leaving out small bowel involvement) and decide upon resectability. In some cases, the diagnostic laparoscopy was carried out by another team in our department and so did not demand a re-laparoscopy through us. After operability was confirmed, laparotomy was performed through a midline incision. As per our technique, in some cases, peritonectomy will be carried out first, starting in the midline and carefully avoiding falling into the abdomen, down the paracolic gutters using the ureters as the posterior limit of the resection. Afterwards, the peritoneum was opened for thorough abdominal exploration and final PCI determination. If ascites were present, a sample was sent in for histological assessment. For upper GI tumors, resection consisted of either a subtotal gastrectomy for distal tumors or a total gastrectomy with or without distal esophagectomy. In both cases, lymphadenectomy was carried out. For GI tumors of the colon or appendix, a left- or right-sided hemicolectomy was carried out and for rectal cancers, an anterior rectal resection was carried out if needed. Additionally, small bowel resections may have also been needed, if the superficial resection of the PD was not possible. Furthermore, cytoreduction consists of omentectomy and any necessary parietal peritonectomies to remove all gross peritoneal disease. Also, some extensive resections may entail liver resections, splenectomies, or partial pancreatic resections, the ultimate goal being to achieve the completeness of cytoreduction. Complete cytoreduction is achieved when there is no apparent peritoneal seeding in the operated field, with a rating of CC-0. CC-1 indicates persistent nodules under 2.5 cm, whereas CC-2 is described as nodules between 2.5 and 5 cm. Finally, CC-3 indicates PD being left behind over 5 cm or unresectable. CC-0 and CC-1 are both considered complete cytoreduction only if perioperative intraperitoneal chemotherapy is applied [5].

Prior to HIPEC, all necessary anastomoses were fashioned, since HIPEC was administered using the closed abdomen technique for 60 or 90 min. The cytotoxic agents used, alone or in combination, were cisplatin (75 mg/m^2^), mitomycin C (15 mg/m^2^), oxaliplatin (270 mg/m^2^), and/or doxorubicin (15 mg/m^2^) according to the tumor entity. All patients were admitted postoperatively to the ICU. After discharge from the ICU, patients were transferred to a dedicated surgical ward for PD patients. Here, they were cared for by two surgical residents, two attending surgeons, a dedicated team of nurses, psychologists specializing in cancer patients, nutritionists, and physiotherapists. Immediately after discharge, the patients were presented again in the MDT to decide on further therapeutic options.

### 2.3. Morbidity and Mortality

Post-operative complications were evaluated within 30 days after surgery and assessed based on Clavien–Dindo’s (CD) classification. All complications were documented by the surgical resident of the ward the day before discharge of the patient and were then reviewed and validated the next day during a department meeting of all attendants, including the chief of the department. Complications were not recorded by the surgeons of the case to remain as impartial as possible. Furthermore, because of the intricacies of the German healthcare system, we are very confident that patients with postoperative complications were readmitted to our hospital or, in those few cases when not, we were in contact with the treating physician and thus were able to collect the respective information. All complications fell under the following categories: gastrointestinal (GI), of cardiac, renal or vascular origin, central nervous system, systemic infection, of pancreatic and liver/bile origin, and bleeding. “Medical” complications were defined as those that were not a direct result of the surgery itself and were classified according to organ. These complications comprised renal, cardiac, infectious, GI, neurological, and pulmonary postoperative events. “Surgical” complications were defined as complications resulting from the trauma of the surgery itself and consequences of intraoperative surgical management of the disease. Our institution considers grave complications as those falling under the CD 3b category or higher because of the need of an intervention under anesthesia or sedation, meaning that a bed-side resolution is not possible. Also, these complications will always prolong hospitalization time. Surgical complications were documented as GI events, postoperative bleeding, surgical site infections (SSI), burst abdomen, fistulae, and anastomotic leakage.

### 2.4. Statistics

All statistical analyses were performed with STATA 18 (StataCorp LLC: College Station, TX, USA). Fisher’s exact test and odds ratios were used to compare the likelihood of complications among k = 2 groups. Uni- or bivariate logistic regression models of complication rates were constructed, including continuous and categorical predictors. Tests were considered statistically significant when the two-sided *p*-value was less than 0.05.

## 3. Results

### 3.1. Population

Between January 2018 and December 2023, there were 305 quality management (QM) surveys of patients, 189 (62%) females and 116 (38%) males, that underwent CRS–HIPEC at our center. The median age at the time of surgery was 58 years (range 19–82) with a median PCI of 11 (0–39; missing data = 16). The patients had peritoneal disease from colorectal cancer (n = 88, 29%), appendiceal disease (n = 77, 25%) including pseudomyxoma peritonei (PMP; n = 23, 30% of appendix, 8% of total), gastric cancer (n = 60, 20%), malignant peritoneal mesothelioma (MPM; n = 34, 12%), ovarian cancer (n = 9, 3%), cancer of unknown primary (CUP; n = 11, 4%), primary peritoneal cancer (n = 8, 3%), small bowel (n = 7, 2%), and other origins (n = 11, 4%). A concurrent disease was present in 65% of the patients (Table 1 and Table 2).

### 3.2. Cytoreductive Surgery

In a dedicated theater, CRS and HIPEC are carried out approximately three times a week. The median time of surgery was 434 min (range of 148–902), including the 60 or 90 min HIPEC regime. Parietal peritonectomy, omentectomy, and cholecystectomy were the most frequently performed organ resections, comprising 79%, 78%, and 75%, respectively. This highlights the fact that a cholecystectomy will be performed almost per principal in our team. In a little over 50% of the cases, a pelvic peritonectomy and/or a right-sided epigastric peritonectomy was performed, followed by a left-sided epigastric peritonectomy in 49% of the cases. A peritonectomy from the mesentery was required in 40% of the cases. Appendectomies and right hemicolectomies were carried out in 32% and 29% of the cases, respectively. Furthermore, an adnexectomy was performed 87 times (29%), a gastrectomy 64 times (21%), an anterior rectal resection 48 times (16%), and a splenectomy 36 times (12%). Lastly, liver resections, esophagus resections and left-sided hemicolectomies were performed in 12%, 9%, and 4% of the cases, respectively. A CC-Score of 0 (CC-0) was achieved in 120 (39%) cases, CC-1 in 124 (41%) cases and CC-2 or above in 39 (13%) cases (missing data = 22 (7%) cases). In regard to the number of anastomoses, in 92 (30%) patients, no anastomoses were fashioned, one was fashioned in 110 (36%) patients, two in 74 patients (24%), three in 14 (5%) patients, four in two patients (1%) and five anastomoses were fashioned in one patient (0.3%) (missing data = 12 (4%) patients). See Table 3 and Table 4 for a summary.

### 3.3. Hyperthermic Intraperitoneal Chemotherapy

As per the standard in our institution, we exclusively used closed-abdomen HIPEC for intraperitoneal chemotherapy. The chemotherapeutic agents of choice were cisplatin (n = 290, 95%) and mitomycin C (n = 266, 87%), followed by doxorubicin (n = 33, 11%) and oxaliplatin (n = 7, 2%). These were used mostly in combination (i.e., cisplatin and mitomycin C for gastric cancer or cisplatin and doxorubicin for colorectal and appendiceal disease). Oxaliplatin was used until 2018 and when in use, patients received bidirectional chemotherapy with intravenous leucovorin und 5-fluorouracil. The dose used for cisplatin was 75 mg/m^2^ and the dose for doxorubicin and mitomycin C was 15 mg/m^2^. HIPEC was carried out for 90 min in 50.6% of the cases and for 60 min in 51.4% of the cases. The length of HIPEC had no influence on the postoperative complication rate, with an odds ratio (OR) for complications of 0.97 (95% CI: 0.55 to 1.70) in the group receiving HIPEC for 90 min (*p* = 1.000).

### 3.4. Postoperative Complications and Mortality

From January 2018 to December 2023, there was a total of 155 patients with surgical and medical complications recorded out of 305 surgeries surveyed (51%). Some patients had more than one postoperative complication; hence, 344 events in 50 categories were recorded, of which 267 were graded 3a or higher in 92 patients. During the aforementioned period, there were 19 (12%) grade 1, 44 (28%) grade 2, 29 (19%) grade 3a, 35 (23%) grade 3b, and 27 (17%) grade 4 complications recorded. One patient (1%—grade 5) died postoperatively in our ITS due to a heart infarct of the posterior wall in 2022 (Table 5). Yearly complication incidences ranged between 44–58%, being higher as more patients were operated on (2018: 20/44 (45%), 2019: 40/70 (57%), 2020: 32/55 (58%), 2021: 15/34 (44%), 2022: 27/58 (48%), 2023: 21/44 (48%)—see Figure 1). There was no statistically significant difference between complications in male (64/116, 55%) and female (91/187, 49%) patients (OR was 1.33 for males, 95% CI: 0.81–2.17, *p* = 0.234). Regarding age, when dividing into three groups—18–50 (38/86, 44%), 50–70 (86/176, 49%), and over 70 years old (31/43, 72%)—the latter group was significantly more prone to complications (*p* = 0.008). Furthermore, we observed no effect of complication versus age and gender (OR 0.987, 95% CI: 0.95–1.02). The median hospital length of stay was 13 days when no complications occurred in comparison to 19 days (3–164 days) when patients had an uneventful course (*p* < 0.0001). One hundred and ninety-two patients (63%) received neoadjuvant chemotherapy. In this subgroup of patients, the incidence of complications was 47% (91/192 patients) with an OR for complications after receiving neoadjuvant chemotherapy of 0.69 (95% CI: 0.42–1.13, *p* = 0.119).

### 3.5. Surgical Complications

Surgical site infections were the most common postoperative surgical complication with an incidence of 35% (n = 54). The grading followed the CDC guidelines for SSI after surgery (SSI 1–3). Incidences of 21% (n = 32) for SSI-1, 5% (n = 8) for SSI-2, and 9% (n = 14) for SSI-3 were reported. Twelve patients (8%) had a burst abdomen (middle line dehiscence) and required re-operation. The incidence of anastomotic leakage (AL) was 5% (n = 8), with no events between 2021 and the present. The formation of fistulae occurred in 2% (n = 3) of cases, two of which were enterocutaneous fistulae formed (ECF) by long-standing intra-abdominal drainage tubes. Furthermore, 2% (n = 3) had a GI hemorrhage and one patient (<1%) had a GI ischemia. A postoperative hematoma was registered in 3% (n = 5) of the cases, and acute postoperative bleeding only in 2% (n = 3). Two patients (1%) had postoperative pancreatic fistulas (POPF) grade B. One patient had a bile duct leakage (<1%), and one patient (<1%) had a leakage of the duodenal stump. A total of 44 patients (44/305, 14%) had to be re-operated due to postoperative complications. Postoperative complications were more frequent in patients with longer surgery times, with an OR of 1.34 per additional hour of surgery (95% CI: 1.20–1.50, *p* < 0.001).

### 3.6. Medical Complications

As for medical complications, the most common complications were of pulmonary origin (59%, n = 91), beginning with pleural effusion (22%, n = 34) and followed by pneumonia (14%, n= 22). Pulmonary embolisms accounted for 7% (n = 11) of all complications. Other pulmonary complications were pneumothorax (5%, n = 8) and respiratory failure (7%, n = 11), of which three patients (2%) required re-intubation, while the rest could be treated with non-invasive techniques and treatments. After pulmonary complications, GI complications were the second most common complication (only CD > 3: 30%, n = 46), with most being gastroparesis (12%, n = 18) followed by perforations (5%, n = 8). With a 2% (n = 3) incidence rate, GI bleeding followed. The least common GI complications were ileus (1%, n = 2) and ischemia (<1%, n = 1). Complications categorized under renal origin had an incidence rate of 35% (all CD, n = 54), the most common being urinary tract infections with an incidence rate of 19% (n = 29, CD > 3 7%, n = 11) followed by acute kidney injury at 9% (n = 14, CD > 3 6%, n = 10), of which only two patients (1%) needed dialysis. Pertaining systemic infections, seven (5%) patients suffered from sepsis and two (1%) patients from systemic mycosis.

Further noteworthy complications were deep venous thrombosis, stroke, and delirium with 2% (n = 3) each, two patients with a grade B pancreatic fistula (1%), and finally cystic duct leakage, liver abscess, and bilioma in one patient (<1%) each. All medical and surgical complications are documented in detail in Table 6.

## 4. Discussion

This retrospective study set out to objectively and critically evaluate the postoperative complications of our surgical department’s center for peritoneal surface malignancy and its team. Cytoreductive surgery and HIPEC is probably the most extensive abdominal surgery performed currently, as it aims to remove all visible tumor implants within the abdomen. As such, it may only be performed by highly trained surgical oncologists with a specific set of skills, as CRS entails multi-visceral resections and extensive peritonectomy. In entities like PMP and some peritoneal metastasized diseases, CRS may take up to 14 h, followed by another 60 or 90 min of perfusion time. This not only makes it probably the most major abdominal surgery carried out but also poses a challenge for the surgeon, anesthesiologist, and afterward, the intensive care doctor. Careful consideration of intraoperative fluid management is just as crucial to patient safety as a safe anastomosis. The morbidity of this treatment is expected to be high and as such, complication prevention and optimal management demand our attention. In 2017, we started systematically documenting all postoperative complications that occurred in our department using the CD classification [6]. The advantage of this classification lies in its simplicity and its general applicability to surgical procedures. To ensure the broadest possible applicability, the classification was based solely on the severity of complications and the extent of their treatment, rather than an assessment of individual organ systems. This approach aims to avoid the downgrading of complications due to the subjective evaluation of organ damage and their causes in retrospective analyses [4]. For this study, we limited the postoperative window of complications to 30 days. In our experience, it is in this timeframe where the most serious complications might occur and where we currently concentrate our efforts the most.

A complication management documentation tool was created inside our hospital’s medical information system, which is filled at the time of discharge by the ward’s surgical resident and is validated the next day during the morning meeting with the attendings and the head of the department. Monthly reports are generated and discussed in our department’s morbidity and mortality (M&M) conferences and quarterly in our team’s own M&M conference, where we also discuss needed changes to SOPs and pay special attention to new problems. These meetings, in analogy to our multidisciplinary tumor boards (MDT), are also composed of various medical specialists and healthcare workers, who share their experiences with our patients and recommend solutions. From 305 surveyed surgeries, 92 patients (30%) had a CD grade < 3b for postoperative complications and 63 patients (20.6%) had a CD grade ≥ 3b for adverse events. Forty-nine percent of patients were discharged without complications.

After major abdominal surgery, some of the most devastating surgical complications are duodenal leakages, pancreatic fistulas, and AL, as it worsens outcomes, increases times and costs of hospitalization, and can ultimately be fatal [7]. The literature reports a wide incidence rate of AL of 2.8–30%, 75% of which are of rectal origin [7,8,9]. These cases are associated with a postoperative mortality rate of up to 22% [9,10]. Our team adheres to the department standard of carrying out side-to-side, isoperistaltic, double-layered hand-sewn anastomosis, as this has shown a low AL rate across all teams in our department. In our cohort, the incidence of AL was 2% with eight ALs from 313 confectioned anastomoses. These were two descendorectostomies and esophagojejunostomies each, one transversorectostomie, one gastroenterostomie, one AL from the duodenal stump, and one ileoileostomie. The AL rate of rectal origin ended in diverting stomas, one of the esophagojejunostomies could be treated with endoscopic clipping and the rest were oversewn. Our last documented AL was in 2020, when we had three cases. In 2018, our group [11] reported on AL rates after extraperitoneal rectal resections without a loop ileostomy. With an incidence rate of 2.1% (1/53 patients), we concluded that ileo- and colorectal anastomosis could be fashioned, and loop ileostomies avoided if carried out in patients with a low risk of AL in experienced centers. Other groups have also published low AL rates by implementing an understanding of the pathophysiology behind AL, carefully selecting patients and using a systematized approach. Barrios et al. [12] from Barcelona even postulate that near-zero AL rates in CRS and HIPEC could be achievable if the forementioned factors are taken into consideration. They reported an AL incidence rate of 0.7% in 1107 anastomoses. A logistic regression showed that the duration of surgery also had an impact on postoperative complications, specifically that with every extra hour of surgery, the OR increased 1.3-fold. This was also corroborated in a 2018 meta-analysis from Cheng et al. that showed the odds for complications were two times greater in general surgery operations lasting longer than 3.8 h [13].

Surgical site infections (SSIs), as classified by the CDC [14], were the most prevalent postoperative complication. A finding consistent with the extended duration of these procedures, which typically exceed five hours and subject tissues to prolonged stress. During wound closure, meticulous attention was given to prevent dehiscence. The fascia was sutured bidirectionally using three or four absorbable polydioxanone sutures, and the wound was thoroughly irrigated with an antiseptic solution prior to skin closure. Despite these precautions, the incidence rate of SSIs remained significant at 35%, with 12 (3.9%) patients experiencing fascial dehiscence (burst abdomen), requiring emergency surgical intervention. The literature is scarce, but some authors describe a prevalence of burst abdomen up to 11% after major abdominal surgery [15,16]. Observations made in this study have led us to implement primary prophylactic wound vacuum therapy in selected high-risk patients. We have not yet appraised the impact of this measure, though. Furthermore, we have been able to reduce the prevalence of superficial incisional SSIs (SSI-1) over the last 5 years.

Delving into the patients with GI perforations or fistulae complications, we encountered that a strict classification under medical or surgical complication was difficult to declare. Two out of eight patients with documented GI perforations were due to stomach ulcers and were as medical complications declared. In the remaining three patients, a perforation could not be found. Two patients underwent open surgery with the suspicion of either an AL or a perforation and neither were intraoperatively confirmed. The third had various endoscopies under the suspicion of an AL of the esophagojejunostomy but was never confirmed, so a microperforation was diagnosed and documented. Ultimately, these complications were some of the most difficult to treat and where we made use of all assets at our disposal.

Intraperitoneal chemotherapeutics, especially platin-based ones, will have treatment-related toxicities. Although the chemotherapeutic agents used show a higher area under the curve (AUC) ratio in peritoneal fluid than in plasma [17], particularly when heated or pressurized [18], they are still able to permeate the tissue barrier into the bloodstream and cause, among others, acute kidney injury. Furthermore, chronic kidney injury can occur in up to 7% of patients after complete CRS and HIPEC [19]. In our cohort, a total of 14 patients presented an acute kidney injury (9% of all complications) and since implementing the use of sodium thiosulfate as an intra- and postoperative nephroprotector into our SOP, no patient has had the need for dialysis. This measure, described in our institution by Kurreck et al. [20], was a direct consequence of the strict observation of our center’s postoperative complications.

Pulmonary complications also played an important role in our cohort. Patients with extensive diaphragmatic peritonectomy that were at risk of plural effusion received an intraoperative chest tube. An important difference to be made is between patients that received a metachronous chest tube as a postoperative complication of pleural effusion or pneumothorax and patients that had a differed chest tube placement in the first postoperative days. Seven of eight patients with pneumothorax received a chest tube to manage the complication. A deeper look into our records shows that only 16 patients out of 35 with pleural effusion needed a chest tube to manage the pleural effusion (documented as over 3b). Our documentation system only lets us extrapolate that those with CD grades under 3b most likely were those with deferred chest tube placement and as such could be excluded as a postoperative complication. This is a limitation of our documentation tool and we are exploring definitions to set a threshold as to when the placement of chest tubes is due to a complication or is to be expected after extensive surgery and inserted for symptomatic relief. A common and grave complication after major abdominal surgery are thromboembolic events. In our cohort, pulmonary embolisms (PE) carried an important significance at a 7% incidence rate. All patients were re-admitted to the ICU and had a prolonged hospital stay. Further inquiry into these cases showed that all patients had received perioperative thrombosis prophylaxis as per our departmental SOP. Furthermore, no evident variations in usual fluid management or coagulopathies were apparent. Although not statistically significant, a clear trend showed that most PE occurred in patients with appendiceal malignancy. Some other factors like surgery duration, patient positioning, and transfusions of blood products were also excluded.

Complications linked to infections also played a significant role in our cohort. Fourteen percent of events were hospital-acquired pneumonias (HAP), being as high as 50% of all pulmonary complications in two consecutive years. In the limited amount of the literature available, postoperative HAP has an incidence rate of 2–29% [21], being the third most common postoperative infection after wound infections and urinary tract infections (UTIs). In our cohort, UTIs were responsible for 19% (n = 29) of postoperative complications. Furthermore, seven patients (5%) had postoperative sepsis and two with fungal infections that led to mycosis.

As evidenced, complications have to be expected after major surgery and as such, decisive and timely action must be taken. Managing complications requires multidisciplinary action and experience. Five percent (n = 14) of our patients needed endoscopic resolution of their complication, and in one patient, an angiography was carried out to stop postoperative bleeding, which could then be drained via a computer tomography-guided drainage. This was one case out of twenty-nine (10%) where complications could be managed with an interdisciplinary and minimally invasive approach.

## 5. Limitations

This was a single-center observational study of a reference center for peritoneal surface malignancy in Germany. As such, the observations herein cannot be extrapolated to other specialized centers in other countries and cannot be externally validated. Nevertheless, the databases used are prospectively maintained and managed by trained, full-time personnel and revised by medical personnel. Also, it is precisely the point of this study to have a critical look into our own complication incidences to better understand them and act upon them. It is in the best interest of this study to avoid observer bias or failure in reporting standards; thus, the documentation tools were not filled out by the operating surgeon. Furthermore, the study is tailored to a very specific patient population, meaning that there is an intrinsic selection bias. Additionally, 30-day morbidity and mortality may well be too short a span of time to detect late postoperative complications.

## 6. Conclusions

Major abdominal surgeries such as CRS and HIPEC come at a cost to the patient. Even when complete cytoreduction is achieved and the best possible oncological outcome may be attainable, the inevitable fact remains that the associated morbidity is high. Strict surveillance and transparency regarding postoperative complications in large reference centers is key to minimize the risk for patients. Scrutinizing our numbers and questioning our decision-making has brought forth a novel approach to complication management in our department. As a university hospital and national reference center, we have a great deal of assets that are available to us and as such, we are able to resolve many complications in a minimally invasive manner. Furthermore, in a short span of time, we have conducted, and will continue to conduct, studies in which the main objective is either the prevention of complications or the acute treatment of them via means other than the surgical. Quality management programs in our department have led to tangible results in our efforts to offer the highest standard of care without compromising patients’ safety.

## Figures and Tables

**Table 1:** Patient characteristics of 305 cytoreductive surgeries in the timeframe of January 2018 to December 2023. LOS: length of stay, including intensive care unit stay.

Patient Characteristics		
Age (median, range)	58 years	19–82
Females	189	62%
Males	116	38%
Concurrent disease (yes)	198	65%
Neoadjuvant chemotherapy (yes)	192	63%
LOS (median, range)	13 days	3–148

**Table 2:** Origin of primary tumor. PMP: pseudomyxoma peritonei; CUP: cancer of unknown primary; MPM: malignant peritoneal mesothelioma.

Origin		N		%	
Colon		88		29%	
Appendiceal	PMP	77	23	25%	8%
Gastric		60		20%	
MPM		34		11%	
Ovarian		9		3%	
CUP		11		4%	
Primary peritoneal		8		3%	
Small bowel		7		2%	
Other		11		4%	

**Table 3:** Number of organ resections in 305 cytoreductive surgeries. Per: peritonectomy.

Organ Resection	N	%
Omentum	238	78%
Per—parietal	242	79%
Cholecystectomy	230	75%
Per—pelvis	161	53%
Per—right epigastrium	159	52%
Per—left epigastrium	149	49%
Per—mesenterium	122	40%
Appendix	99	32%
Right hemicolectomy	89	29%
Adnexa	87	29%
Stomach	64	21%
Small bowel	60	20%
Hysterectomy	49	16%
Anterior rectum	48	16%
Spleen	36	12%
Liver	36	12%
Esophagus	28	9%
Left hemicolectomy	11	4%
Sigma	10	3%
Bladder	9	3%
Pancreas	1	0.3%

**Table 4:** General characteristics of cytoreductive surgery with hyperthermic intraperitoneal chemotherapy. PCI: peritoneal cancer index (Sugarbaker). CC: cytoreduction score.

CRS and HIPEC	N	Percentage (%)
PCI (median, range)	11	0–39
Surgery time (median, range)	435 min	148–902 min
CC-Score		
CC-0: no disease	120	39%
CC-1: <2.5 mm	124	41%
CC-2: 2.5–25 mm	23	8%
CC-3: >25 mm	16	5%
Missing data	22	7%
N of anastomoses		
0	92	30%
1	110	36%
2	74	24%
3	14	5%
4	2	1%
5	1	0.3%
Missing data	12	4%
Intraperitoneal chemotherapy		
Cisplatin	290	95%
Mitomycin C	266	87%
Doxorubicin	33	11%
Oxaliplatin	7	2%

**Table 5:** Percentage of complications (Clavien–Dindo). N = 155.

Clavien–Dindo Classification	N	Percentage (%) of All Events
1	19	12%
2	44	28%
3a	29	19%
3b	35	23%
4a	26	17%
4b	1	1%
5	1	1%
**Grouped**	**N**	**Percentage (%) of All Events**
1–2	63	41%
>3	92	59%

**Table 6:** Postoperative medical and surgical complications. The percentages shown to illustrate complication rates were calculated as the number of events divided by the total number of patients that presented an event, meaning that the percentages only relate to a part of the cohort.

Postoperative Complication	N	%
Pulmonary		
Pleural effusion	34	22%
Pneumonia	22	14%
Pulmonary embolism	11	7%
Respiratory failure	11	7%
Pneumothorax	8	5%
Re-intubation	3	2%
Empyema	2	1%
**Total events**	**91**	**59%**
Renal		
Urinary tract infection	29	19%
Acute kidney injury	14	9%
I.V. fluid therapy	4	3%
Dialysis	2	1%
Electrolyte imbalance	2	1%
Pyelonephritis	1	1%
Ureter leakage	1	1%
Urinoma	1	1%
**Total events**	**54**	**35%**
Gastrointestinal		
Gastroparesis	18	12%
Perforation	8	5%
Anastomose leakage	8	5%
Fistulae	3	2%
GI bleeding	3	2%
Stoma	3	2%
Ileus	2	1%
Ischemia	1	1%
**Total events**	**46**	**30%**
Systemic infection		
Bacterial	9	6%
Sepsis	7	5%
Mycosis	2	1%
Clostridium difficile	1	1%
Fungal	1	1%
**Total events**	**20**	**13%**
Neurological		
Delirium	3	2%
Stroke	3	2%
**Total events**	**6**	**4%**
Cardiovascular		
Deep vein thrombosis	3	2%
Infarct	1	1%
Arrythmia	1	1%
**Total events**	**5**	**3%**
Hepatobiliary		
Cysticus leakage	1	1%
Liver abscess	1	1%
Bilioma	1	1%
**Total events**	**3**	**2%**
Surgical Site Infections		
SSI-1	32	21%
SSI-2	8	5%
SSI-3	14	9%
Burst abdomen	12	8%
**Total events**	**54**	**35%**
Hematological		
Hematoma	5	3%
Post-op bleeding	3	2%
**Total events**	**8**	**5%**

## Data Availability

Due to German and European Data Protection regulations, the data are not publicly available. Further questions may be directed to the authors.
